# Evaluation of the effect of an extract of *Sphingomonas xenophaga* present in a Thermal Spring Water in the management of sensitive skin associated with cutaneous vascular disorder

**DOI:** 10.1111/ics.13087

**Published:** 2025-06-22

**Authors:** Pascal Hilaire, Carine Ballihaut, Celine Cornillon, Mark Donovan, Cosima Dufour‐Schroif, Delphine Kerob, Jean‐Jacques Schoonjans, Anna Veriato

**Affiliations:** ^1^ L'Oreal Research and Innovation Notre Dame d'Oé France; ^2^ L'Oreal Research and Innovation Aulnay‐sous‐Bois France; ^3^ L'Oreal Research and Innovation Chevilly la Rue France; ^4^ La Roche Posay Laboratoire Dermatologique Levallois‐Perret France; ^5^ Novéal Le Thillay France

**Keywords:** *Sphingomonas* extract, telangiectasia, vascular disorder

## Abstract

**Objective:**

La Roche Posay Thermal Spring Water (LRP TSW), which contains specific minerals and possesses a unique microbial composition, has proven anti‐oxidant, anti‐inflammatory, pre‐ and post‐biotic properties and an ability to improve skin barrier function. Our objective was to confirm the effectiveness of a biomass isolated from LRP TSW on inflammatory and vascular parameters in sensitive skin.

**Methods:**

A fully characterized strain of *Sphingomonas xenophaga* was isolated from LRP TSW. An industrial fermentation process was developed to obtain a reproducible biomass (the ‘ferment extract’) in order to evaluate its effect on skin parameters in vitro and in vivo. Inhibition of pre‐kallikrein activity, which converts pro‐bradykinin into inflammatory vasoactive bradykinin, by the ferment extract was determined in vitro. In vivo, the effect of a 4‐week, twice‐daily application of a 2% ferment extract cream formulation on vascular disorders was investigated in a randomized study including 86 Caucasian female subjects presenting permanent redness and vascular disorder on the face in comparison to the cream vehicle.

**Results:**

The ferment extract inhibited in a dose‐dependent manner pre‐kallikrein activity in vitro, inducing 46% and 97% inhibition at concentrations of 0.4% and 0.5%, respectively. In vivo after 28 days of twice‐daily applications of the ferment extract and vehicle, both treatments induced a significant decrease in vascular disorder as evaluated by clinical scoring (Dermascore® device with cross‐polarized light). The mean decrease in vascular disorder score from baseline was significantly greater (*p* < 0.05) in the ferment extract group (−0.36) when compared to vehicle (−0.18). In addition, 60% of subjects in the group treated with the formulated ferment extract had a decreased score compared to 33% for the group treated with the vehicle.

**Conclusion:**

Inhibition of the production of inflammatory vasoactive bradykinin by the ferment extract observed in vitro is in line with the anti‐inflammatory effects of the formulated extract as shown in subjects with facial vascular disorder. The results in this study suggest that this ferment extract is a potentially new active ingredient that could be used either alone or in combination with other soothing agents to target skin inflammatory pathways and to improve skin vascular disorder.

## Graphical abstract

Inhibition of the kallikrein‐kinin system is a potential approach to the dermocosmetic treatment of skin disorders associated with redness. We showed that a cream formulation with a *Sphingomonas* ferment extract inhibiting this system was effective in improving vascular disorder after repeated application in female volunteers with sensitive and reactive skin.

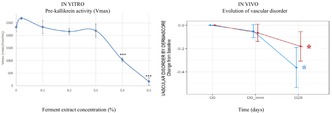

## Résumé

**Objectif:**

L'eau thermale de La Roche Posay (ET LRP), qui contient des minéraux spécifiques et possède une composition microbienne unique, a des propriétés antioxydantes, anti‐inflammatoires, pré‐ et post‐biotiques prouvées, et une capacité à améliorer la fonction de barrière cutanée. Notre objectif était de confirmer l'efficacité d'une biomasse isolée de l'ET LRP sur les paramètres inflammatoires et vasculaires d'une peau sensible.

**Méthodes:**

Une souche entièrement caractérisée de *Sphingomonas xenophaga* a été isolée à partir de l'ET LRP. Un processus de fermentation industrielle a été mis au point pour obtenir une biomasse reproductible (l’« extrait de ferment »), afin d'évaluer son effet sur les paramètres cutanés in vitro et in vivo. L'inhibition de l'activité de la pré‐kallikréine, qui convertit la pro‐bradykinine en bradykinine vasoactive inflammatoire, par l'extrait de ferment a été déterminée in vitro. In vivo, l'effet d'une application biquotidienne de 4 semaines d'une formulation de crème à 2% d'extrait de ferment sur les affections vasculaires a été étudié dans une étude randomisée incluant 86 femmes caucasiennes présentant des rougeurs permanentes et des affections vasculaires sur le visage, en comparaison avec la crème véhicule.

**Résultats:**

L'extrait de ferment a inhibé de manière dose‐dépendante l'activité pré‐kallikréine in vitro, induisant une inhibition de 46% et 97% à des concentrations de 0,4% et 0,5%, respectivement. In vivo après 28 jours d'application deux fois par jour de l'extrait de ferment et du véhicule, les deux traitements ont induit une diminution significative des troubles vasculaires, telle qu'évaluée par le score clinique (dispositif Dermascore® à lumière polarisée croisée). La diminution moyenne du score d'affections vasculaires, par rapport au début de l'étude, était significativement plus importante (*p* < 0,05) dans le groupe extrait de ferment (−0,36) que dans le groupe véhicule (−0,18). De plus, 60% des sujets du groupe traités avec l'extrait de ferment formulé avaient un score diminué par rapport à 33% pour le groupe traité avec véhicule.

**Conclusion:**

L'inhibition de la production de bradykinine vasoactive inflammatoire par l'extrait de ferment observé in vitro est en ligne avec les effets anti‐inflammatoires de l'extrait formulé comme montré chez les sujets atteints d'affections vasculaires faciales. Les résultats de cette étude suggèrent que cet extrait de ferment est un principe actif potentiellement nouveau qui pourrait être utilisé seul ou en association avec d'autres agents apaisants pour cibler les voies inflammatoires de la peau et améliorer les affections vasculaires cutanées.

## INTRODUCTION

Rosacea is a chronic inflammatory skin disease where vascular abnormalities are frequent and can manifest as permanent redness, associated or not with hot flushes. The erythema associated with rosacea in particular represents a complex clinical challenge and likely has a multifactorial aetiology. Contributing factors include neurovascular dysregulation, increased levels of pro‐inflammatory mediators and aberrant vasodilation. The activation of transient receptor potential (TRP) channels at cutaneous sensory nerve endings triggers the release of vasoactive peptides, driving neuroinflammation and resulting in redness, burning and stinging sensations [1, 2]. Erythema of rosacea causes a marked decrease in Health‐Related Quality of Life in most patients, although there are still unmet needs to improve this clinical sign with effective and safe treatments [3].

Overall, facial cutaneous vascular disorders are a broad spectrum of disorders including hemangiomas, couperosis and rosacea. Their pathophysiology and treatment are specific. In case of redness associated with rosacea, a recent systematic literature review analysed the different treatment options, the most well‐studied treatments including light‐based therapy (intense pulse light, pulsed dye laser), topical treatments such as metronidazole, azelaic acid, oxymetazoline and brimonidine tartrate [1]. Couperosis is mainly treated with light‐based therapies.

Our study was a unique approach to assess the mode of action and the benefits on vascular disorders associated with rosacea of a biomass of *Sphingomonas xenophaga*. The specific mineral and microbial compositions of La Roche‐Posay Thermal Spring Water (LRP TSW) have been studied in vitro and in vivo, highlighting its benefits in improving skin barrier, inflammation, oxidative stress and microbiome as well as dermatological conditions including dry skin, atopic skin and psoriasis [4]. Use of bacterial extracts of bacteria has shown to improve some skin conditions: topically applied extracts like *Lactobacillus reuteri* [5] and gram‐negative bacterium *Vitreoscilla filiformis* improved the symptoms and reduced SCORing Atopic Dermatitis (SCORAD) [6, 7], the latter possibly via augmented antioxidant and antimicrobial defences [8].

The objective of this study was to process a bacterial strain from LRP TSW to obtain an ingredient with interesting biological properties for inclusion in a product for dermocosmetic skin care use. A flagellated bacterial strain of *S. xenophaga* was isolated from the endogenous flora component of LRP TSW and fully characterized [9]. An industrial fermentation process was developed with the ultimate objective to study the impact of the *Sphingomonas* extract on vascular skin parameters in vitro and in vivo.

On the one hand, the present study focused on the in vitro effect of the *Sphingomonas* extract on pre‐kallikrein activity, which converts pro‐bradykinin into the inflammatory vasoactive bradykinin moiety [10]. Kallikreins are a large family of serine proteases that play important roles in tissue homeostasis. In skin, the activity of three major kallikreins, kallikrein 5 (KLK5), 7 and 14, is key to proper epidermal differentiation and *stratum corneum* desquamation. In inflammatory skin conditions, such as atopic dermatitis or psoriasis, a cascade of kallikrein activity, initiated by KLK5, can lead to the release of pro‐inflammatory kinins from kininogens ([11] and references therein). One of these kinins, bradykinin, has been reported to induce itch in atopic lesions and is known to be involved in skin red flushes and vasodilation [10]. Furthermore, the inhibition of KLK5 by ivermectin, an antiparasitic drug, reduced rosacea‐associated inflammation [12].

On the other hand, this study was performed to investigate the potential in vivo effect of the formulated (cream) *Sphingomonas* extract on vascular disorders clinically observed in patients with sensitive and reactive skin (e.g. patients with rosacea) in comparison with the cream vehicle.

## MATERIALS AND METHODS

### Biomass manufacturing process

A culture of the *S. xenophaga* CNCM‐I 5455 strain was carried out in its complete culture medium in an efficient 3000‐L bioreactor in batch mode. During this step, the pH was not regulated, the temperature was maintained at 26°C and the dissolved oxygen at 30%. As soon as the stationary phase was reached, separation of the cells was carried out by centrifugation. The resulting pellet (i.e. the biomass), containing the cells, was recovered, frozen at −20°C and then thawed, allowing the cells to burst and thus obtain a lysate. The lysate was then packaged in bags and finally stabilized by sterilization. The INCI name of this new ingredient is *Sphingomonas* ferment extract (the ‘ferment extract’ hereafter).

### In vitro effect of ferment extract on pre‐kallikrein activity

Enzymatic kallikrein activity was monitored by spectrophotometry (λ = 405 nm) using a chromogenic substrate (L2120 [H‐D‐Pro‐Phe‐Arg‐pNA.2HCl, Cas Number: 4029234], Bachem®, Bubendorf, Switzerland) in the presence of 12.5 μg/mL dextran sulphate, a potent activator of the human plasma kallikrein kinin system [13]. The kinetics of enzymatic activity were monitored and the maximum speed reaction (*V*_max_) was calculated. Results represent the mean ± SD of at least two duplicates from two independent experiments.

In a preliminary test in the absence of plasma pre‐kallikrein and dextran sulphate, the effect of a 0.5% (w/v) concentration of the ferment extract on substrate cleavage was measured by incubation of the substrate with the extract and the absorbance measurement over a 15‐min incubation period.

In a subsequent test, the effect of the ferment extract on plasma pre‐kallikrein activity was investigated following a 10 min incubation of citrated plasma (pool of normal human plasma [Stago, Ref 00539]) with 0.02%, 0.1%, 0.2%, 0.3%, 0.4% and 0.5% (w/v) concentrations of ferment extract at 0°C, followed by pre‐kallikrein activation by dextran sulphate and absorbance measurement. The statistical significance of ferment extract effects in comparison to control (no extract) was determined by the Mann–Whitney test (*α* < 0.05).

### Clinical study of ferment extract effects in sensitive skin patients

This was a double‐blind, randomized, vehicle‐controlled study conducted on two parallel groups of subjects, one treated with a neutral cream formulation (glyceryl stearate—PEG100 stearate—polyoxyethylene 100 stearate) containing 2% (w/w) ferment extract and one treated with the cream vehicle. Inclusion criteria were

**FIGURE 1:**
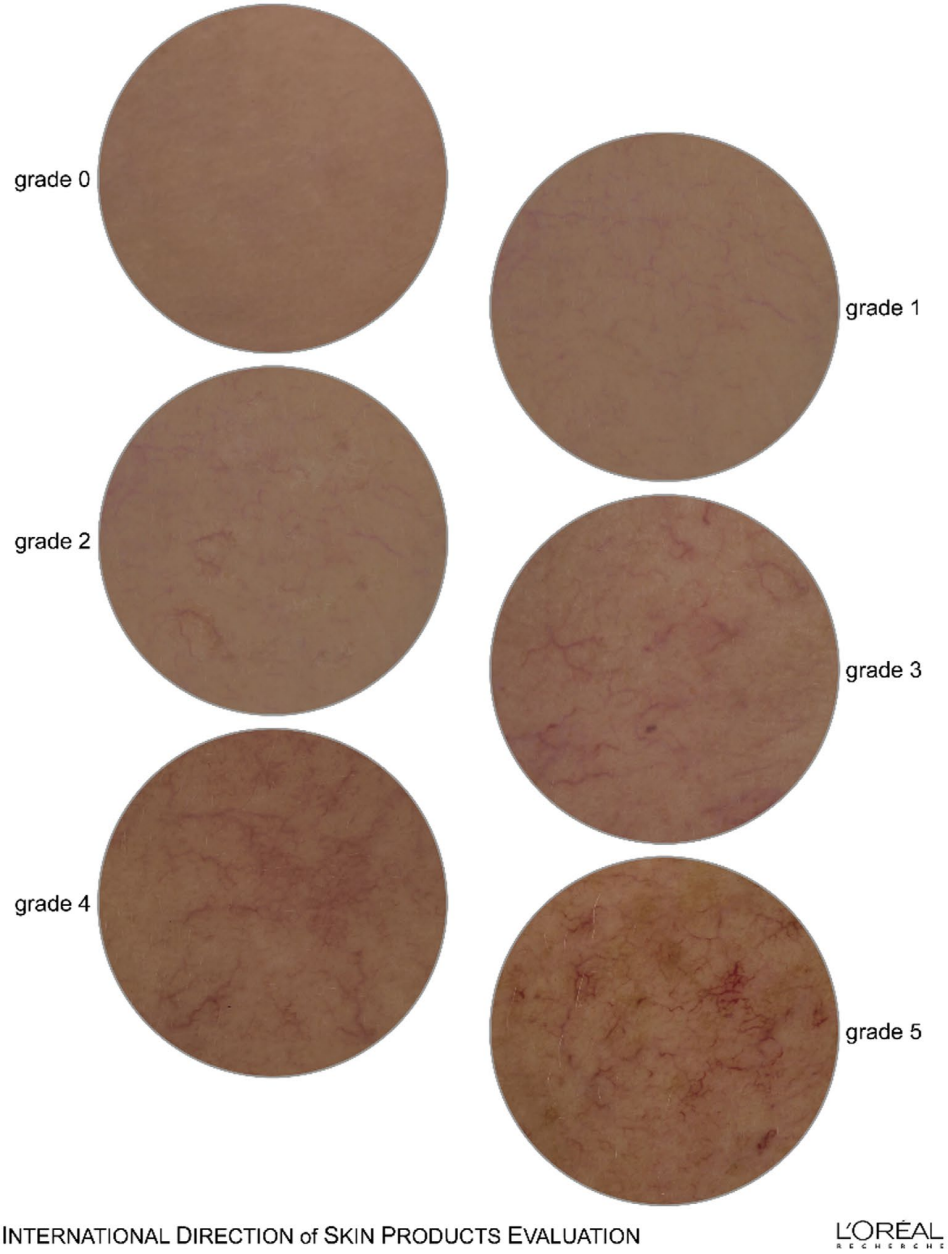
Atlas of the homogeneity of microvascular disorder by Dermascore®.

- Pre‐menopausal or at least 2 years post‐menopausal women
- Congestive hot flushes in case of temperature or climatic changes (hot flushes in relation with pre‐menopause excluded)
- Skin that reacts to environmental conditions (temperature, spicy food, emotional stress with flushes and tightness
- Moderate permanent facial redness (on cheek): diffuse redness on the whole face (grade ≥4 on a 0–9 10‐point scale
- Telangectasia on whole face (intensity grade ≥3 on a 0–9 10‐point scale)
- Vascular disorder quantified with Dermascore® in cross‐polarized light (enabling visualization of the vascular disorder thanks to its angle of polarization), with a score of at least 3 according to the 5‐point photographic referential Caucasian shade L'Oréal Atlas of Micro Vasculature (Figure 1: 0 = no visible microvascular disorder; 5 = very visible micro vascular disorder). Grading the severity of facial skin signs including vascular homogeneity on the basis of referential ethnic group specific Skin Atlases is described in refs [14, 15].

Exclusion criteria were

- Subjects taking medications which, in the opinion of test personnel, might affect test results.
- Subjects with severe rosacea (red bumps, pustules on nose or rhinophyma).
- Subjects having used self‐tan products on the face within the last 4 weeks.
- Subjects having applied any cosmetic products targeting redness or blemishes within the last 4 weeks.
- Subjects having applied cosmetic or pharmaceutical products (other than their usual cleanser) to the face within the 24 h before the day of inclusion.

A consent form was to be signed by all subjects prior to enrolment, including publication of their case details such as photographs. This study was conducted by Alba Science Ltd., Edinburgh, between October and December 2021. As this was a cosmetic study, ethics committee approval was not required. The study was conducted in compliance with the principles of the Declaration of Helsinki 1975, as revised in 1983. No formal claim of Good Clinical Practice (GCP) compliance was required or made for this study; however, the practices and procedures adopted during the conduct of the study were consistent with the Principles of International Conference on Harmonisation (ICH) Guidelines on Good Clinical Practice (CPMP/ICH/135/95).

In order to minimize bias, the study was conducted under randomized conditions with a double‐blinded evaluation by the dermatologist and volunteers. The groups' randomization was to be embedded into the eCRF. The randomization list was drawn up by the Sponsor. A randomization number was attributed at the end of the baseline visit to each subject who had successfully met the criteria for enrolment at the baseline visit. No number was to be omitted or skipped. The randomization number corresponded to the inclusion number allocated to each volunteer.

As per randomization schedule, the following two groups were identified as A and B:

- Group A: Formula # 2039074 03 (ferment extract treatment group)
- Group B: Formula # P203907401 (cream vehicle control group)

Small amounts (penny size) of test products were applied twice daily over 4 weeks on freshly cleansed skin on the whole face avoiding contact with the eyes. Clinical scoring (Dermascore®) was performed by a dermatologist on one cheek as per the randomization schedule, with patients wearing a black cap to avoid a possible influence of head hairs, under standardized conditions of lighting and sitting position (the chair position was marked on the floor). Scoring was performed at baseline before the first application (D0), immediately after the first application (D0_Imm) and at the end of the 4 week application period (D28).

### Statistical analyses

All clinical assessments of efficacy parameters were analysed using a linear mixed model, with

- Y: Change from baseline as a response vector
- X_1: Baseline, X_2: Treatment, X_3: Time and X_4: Treatment–Time interaction as fixed effect
- Intercept per subject as random effect

A continuous first‐order autoregressive model (~1|RD) was used to take into account the repeated measurement by time correlation effect.

Checking the normality of data was performed by using a normal probability plot of model residuals (qqplot) and using a Shapiro–Wilk test at 1%. In case of non‐compliance with the normality hypothesis, a non‐parametric test was preferred (e.g. Wilcoxon test at each time point).

Prior to final modelling, the baseline values homogeneity was checked between the treatment groups for all continuous parameters. If a baseline‐treatment interaction was present, student tests were derived to indicate the value and the strength of the difference. If the difference was weak (none‐weak effect sizes) and not clinically relevant, the final model was run with the baseline‐treatment interaction instead of the baseline term only. If the difference was clinically relevant and with strong effect sizes, only descriptive statistics were communicated in order to avoid the communication of biased results. For the final model, all comparisons between the two groups at each time point and all comparisons of estimated change from baseline with the 0 value were made using contrasts of least square means computed by the mixed model.

Percentages of responders were descriptive values calculated on the basis of a threshold difference of vascular disorder score at Day 28 from baseline value set at 0.2, and no effect size was determined for this parameter.

## RESULTS

### Pre‐kallikrein activity

#### Effect of ferment extract on L2120 substrate cleavage

In the absence of plasma pre‐kallikrein and dextran sulphate, the ferment extract at a concentration of 0.5% did not induce substrate cleavage over the 15 min test period. Of note, at this 0.5% concentration, the extract had a constant absorbance at 405 nm, which indicated that for all subsequent measurements, intrinsic ferment absorbance at this specific wavelength had to be determined and measured absorbance values had to be corrected accordingly.

#### Inhibition of plasma pre‐kallikrein by the ferment extract

**FIGURE 2:**
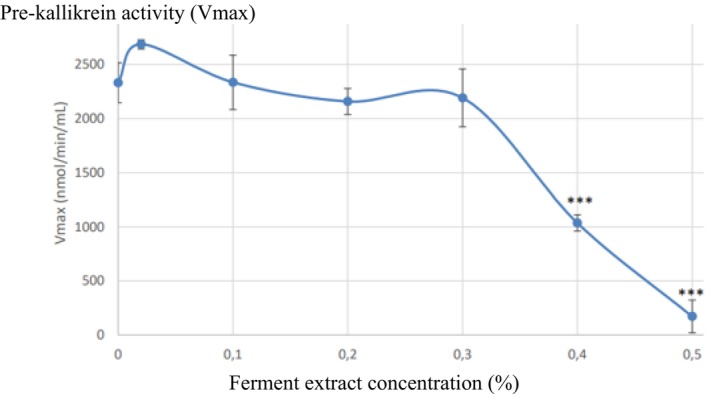
Effect of a 10‐min pre‐incubation of normal citrated plasma with the ferment extract at 0°C on subsequent dextran sulphate‐activated pre‐kallikrein. ***Statistically significant inhibition (*p* < 0.05).

After a 10 min incubation of normal citrated plasma with 0.02%, 0.1%, 0.2%, 0.3%, 0.4% and 0.5% concentrations of ferment extract at 0°C, followed by pre‐kallikrein activation by dextran sulphate, enzymatic pre‐kallikrein activity was found to be inhibited in a dose‐dependent manner, statistically significant 46% and 97% inhibition being observed with the 0.4% and 0.5% concentrations of ferment extract, respectively (Figure 2).

#### Effect of ferment extract application on vascular disorders of subjects with sensitive skin

**FIGURE 3:**
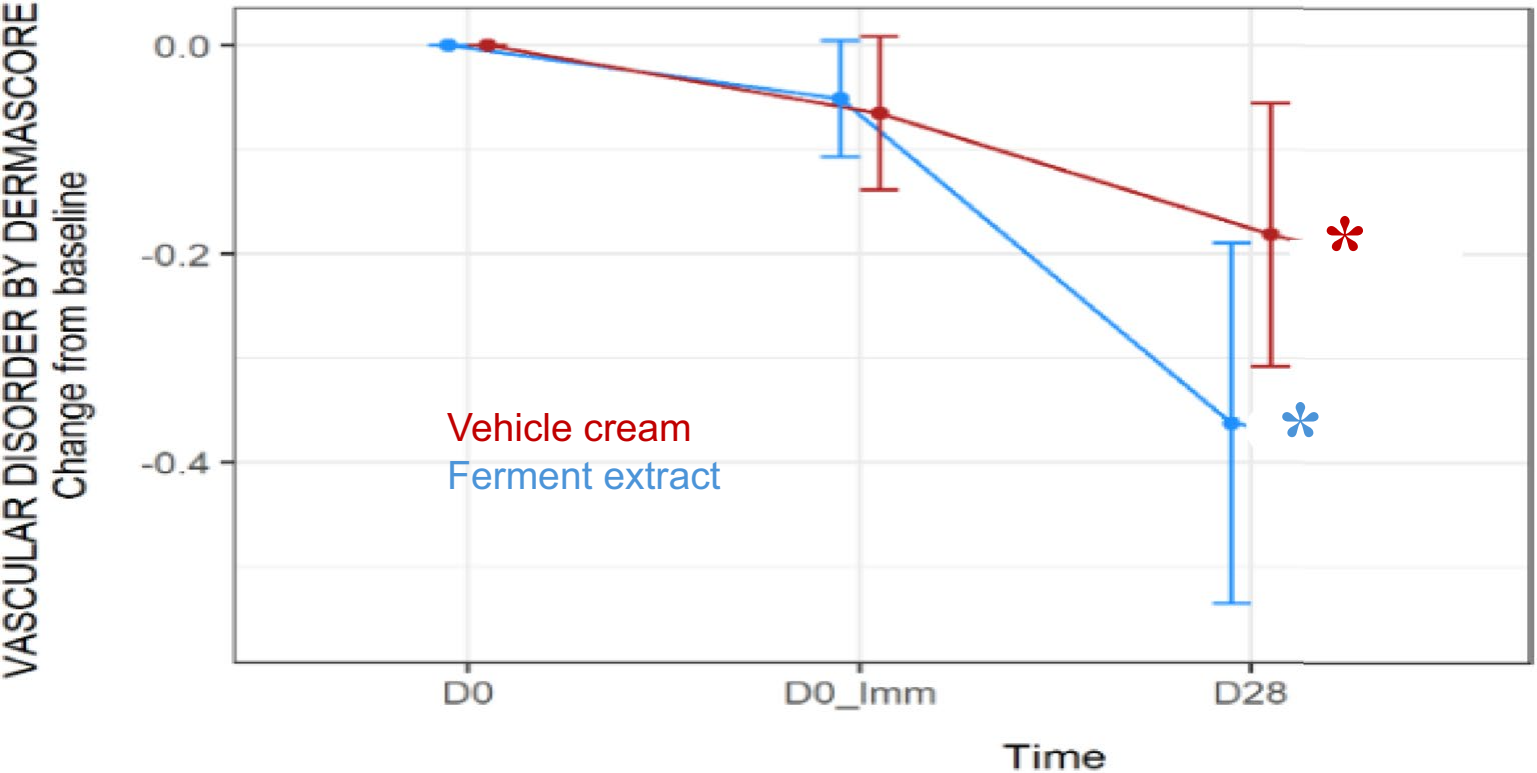
Evolution over time of vascular disorder Dermascore® change from baseline (mean change ± 95% confidence interval). *Statistically significant decrease compared to baseline (adjusted *p*‐value < 0.05).

Eighty‐six Caucasian female volunteers aged from 25 to 65 years, phototypes I to III, with sensitive and reactive skin were recruited in this study and randomly assigned to two treatment groups of 43 subjects (2% ferment extract cream group and cream vehicle group). Clinical scoring as quantified with Dermascore® at baseline before the first application (D0) was (mean ± SD) 3.03 ± 0.77 for the ferment extract group and 3.10 ± 0.86 for the cream vehicle group. After 4 weeks of treatment, both treatments showed a significant reduction in clinical scoring (Table 1). A total of 60% of subjects had a decreased score in the ferment extract cream group, as opposed to only 33% in the cream vehicle group. Overall, the score reduction in comparison to baseline was statistically significant (*p* < 0.05) in both groups (Figure 3).

**TABLE 1:** Mean change of vascular disorder Dermascore® from baseline.

Treatment	Scoring time	*N*	Mean change from baseline	SD	Min	Max	% evolution from baseline
Ferment extract	D0_Imm	43	−0.05	0.18	−0.6	0.4	−2
	D28	43	−0.36	0.56	−1.6	1.0	−10
Cream vehicle	D0_Imm	43	−0.07	0.24	−1.0	0.4	−2
	D28	43	−0.18	0.41	−1.0	0.6	−5

*Note*: D0_Imm, immediately after first product application; D28, at 4 weeks of treatment; max, maximum; min, minimum; *N*, number of subjects; SD, standard deviation.

Inter‐group comparisons at D0_Imm and D28 showed that the amplitude of score reduction on D28 was significantly greater in the ferment extract cream group compared to the cream vehicle group (Table 2).

**TABLE 2:** Intergroup comparisons of Dermascore® decrease immediately after the first application and after 4 weeks of treatment.

Comparison	*N*	Scoring time	Estimate	Lower	Upper	*p*‐Value adjusted
Ferment extract vs. cream vehicle	43 vs. 43	D0_Imm	0.002	−0.162	0.166	0.983
	43 vs. 43	D28	−0.194	−0.358	−0.029	0.038

*Note*: D0_Imm, immediately after first product application; D28, at 4 weeks of treatment; *N*, number of subjects per group. Adjustment: Benjamini–Hochberg.

In terms of tolerance to treatment, no adverse events were reported in the ferment extract group. An event of moderate severity was observed in one subject of the cream vehicle group, showing symptoms of dryness, itching, redness and stinging.

## DISCUSSION

Activating the kallikrein/kinin system leads to the transformation of the inactive proenzymes factor XII and pre‐kallikrein into their active forms: activated factor XII and kallikrein [16]. The increase in quantity and the magnitude of biological activity of KLK5 leads to the increased production of cathelicidins such as LL‐37. LL‐37 is an antimicrobial peptide that increases innate cutaneous inflammation, vasodilation and vascular proliferation, which are underlying pathogenic features of rosacea [17]. Kallikrein 5 cleaves the high‐molecular weight kininogen and releases bradykinin [17]. Bradykinin and its active metabolite desArg(9)bradykinin bind to receptors B2 (RB2) and B1 (RB1), respectively [18]. The interaction of the ligands bradykinin and desArg(9)bradykinin with their respective RB2 and RB1 receptors leads to vasodilation and increased vascular permeability, resulting in water exiting the vessels to the tissues and possible diapedesis of the cells. Intradermal challenge with incremental doses (0.5, 5 and 50 nmol) of either the B1‐agonist desArg(9)‐bradykinin, the B2‐agonists bradykinin or kallidin has been shown to induce a dose‐dependent increase in wheal and flare areas in both atopic and non‐atopic subjects [19]. Bradykinin is therefore involved in vasodilation and an increase in vascular permeability, thus making bradykinin antagonists candidates for the dermocosmetic treatment of skin disorders associated with redness. The extract of *Sphingomonas* genus, subject of the present investigation, can be used as an inhibitor of the kallikrein‐kinin system and therefore attenuate the advent of redness. Indeed, it has been shown on a skin model stimulated by capsaicin that using a composition containing an extract of *S. xenophaga* decreases redness [20]. Facial skin redness may be associated with different diagnoses, the most frequent one being rosacea with erythema. Rosacea represents a spectrum of clinical features with the more common presentations characterized by increased blood flow and vasodilation during disease flares, which accentuate central facial erythema, and can be associated with inflammatory lesions. Recently, a randomized controlled split‐face study has shown that a dermocosmetic cream containing *S. xenophaga* extract significantly improved erythema, skin sensitivity, demodex count, QoL and feeling of stigmatization of female with rosacea and sensitive skin, with a very good tolerance [21].

Moreover, in three‐dimensional reconstructed skin models, the *S. xenophaga* biomass has been shown to preserve skin barrier function by significantly increasing the levels of tight junctions‐associated proteins and inhibiting Kallikrein 7 activity, a protease involved in epidermal desquamation [22]. Improvements in the barrier function significantly reduce symptoms of rosacea [23].

Overall, results obtained in the present study have shown that a *Sphingomonas* ferment extract is able to inhibit in a dose‐dependent manner pre‐kallikrein activity in vitro. Because activation of the kallikrein/kinin system results in the release of bradykinin and its active metabolite desArg(9) bradykinin, which are involved in vasodilation and an increase in vascular permeability, it is assumed that inhibition of the kallikrein‐kinin system is a potential approach to the dermocosmetic treatment of skin disorders associated with redness. Indeed, the present investigation demonstrated that, in vivo, after 28 days of twice‐daily application of a cream formulation of the ferment extract in subjects with sensitive and reactive skin, there was a noticeable decrease in vascular disorder as evaluated by clinical scoring, and this decrease was significantly greater than that observed with the cream vehicle alone. These findings are in line with previous observations of *S. xenophaga* anti‐inflammatory activity in skin models and in subjects with rosacea and sensitive skin.

One limitation of the present study might come from its conduct in the immediate post‐Covid period (October to December 2021), and a potential interference of vaccines and masks with facial microvasculature cannot be firmly ruled out.

## CONCLUSION

In the present study, *S. xenophaga* from LRP TSW has shown to have interesting biological properties, both in vitro on the kallikrein‐kinin system and in vivo in subjects with sensitive and reactive skin. These unique properties make this ferment extract a potentially new active ingredient that could be used alone or in combination with other soothing agents to target skin inflammatory pathways and improve intolerant skin vascular disorders such as rosacea.

## CONFLICT OF INTEREST STATEMENT

All authors are employees of L'Oréal.

## Data Availability

The data that support the findings of this study are available from the corresponding author upon reasonable request.
